# *FLI1* and PKC co-activation promote highly efficient differentiation of human embryonic stem cells into endothelial-like cells

**DOI:** 10.1038/s41419-017-0162-9

**Published:** 2018-01-26

**Authors:** Hao Zhao, Yan Zhao, Zili Li, Qi Ouyang, Yi Sun, Di Zhou, Pingyuan Xie, Sicong Zeng, Lingfeng Dong, Hua Wen, Guangxiu Lu, Ge Lin, Liang Hu

**Affiliations:** 1 Institute of Reproduction & Stem Cell Engineering, School of Basic Medical Science, Central South University, Changsha, China; 2 National Engineering & Research Center of Human Stem Cells, Changsha, China; 3 Key Laboratory of Reproductive and Stem Cell Engineering, National Health and Family Planning Commission, Changsha, China; 4 Reproductive & Genetic Hospital of CITIC–Xiangya, Changsha, China

## Abstract

Rationale-endothelial cells (ECs) play important roles in various regeneration processes and can be used in a variety of therapeutic applications, such as cardiac regeneration, gene therapy, tissue-engineered vascular grafts and prevascularized tissue transplants. ECs can be acquired from pluripotent and adult stem cells. To acquire ECs from human embryonic stem cells (hESCs) in a fast, efficient and economic manner. We established a conditional overexpression system in hESCs based on 15 transcription factors reported to be responsible for hematopoiesis lineage. Among them, only overexpression of *FLI1* could induce hESCs to a hematopoietic lineage. Moreover, simultaneous overexpression of *FLI1* and activation of PKC rapidly and efficiently induced differentiation of hESCs into induced endothelial cells (iECs) within 3 days, while neither *FLI1* overexpression nor PKC activation alone could derive iECs from hESCs. During induction, hESCs differentiated into spindle-like cells that were consistent in appearance with ECs. Flow cytometric analysis revealed that 92.2–98.9% and 87.2–92.6% of these cells were CD31+ and CD144+, respectively. Expression of vascular-specific genes dramatically increased, while the expression of pluripotency genes gradually decreased during induction. iECs incorporated acetylated low-density lipoproteins, strongly expressed vWF and bound UEA-1. iECs also formed capillary-like structures both in vitro and in vivo. RNA-seq analysis verified that these cells closely resembled their in vivo counterparts. Our results showed that co-activation of *FLI1* and PKC could induce differentiation of hESCs into iECs in a fast, efficient and economic manner.

## Introduction

Endothelial cells (ECs) line the internal lumen of blood vessel walls and can directly release proteins into the blood stream. These cells are involved in a variety of tissue system functions, including blood pressure control, interactions with immune cells, uptake of nutrients and so on. Moreover, they are ideal candidates for use as vehicles for gene therapy^1^. Thus far, ECs have been isolated from various sources, such as from peripheral blood mononuclear cells^2^, bone marrow mononuclear cells^3^, cardiac progenitors^4^, adipose-derived stem cells^5^ and umbilical cord blood^6^. However, ECs from these adult sources are reported to be difficult to identify, isolate and expand in culture^7^.

Human pluripotent stem cells (hPSCs) include human embryonic stem cells (hESCs) and human-induced pluripotent stem cells (hiPSCs). Due to their capacities for self-renewal and pluripotency, are considered to be an ideal resource for generating an inexhaustible supply of cells for clinical and scientific applications. There are two general approaches for inducing EC differentiation from hPSCs: the common method is to culture hPSCs in suspension medium to form a 3-dimensional aggregate called an embryoid body and ECs (2–20%) subsequently emerge from the mesoderm after 10–15 days^8^. Another method is to co-culture human ES cells on stromal cells. For example, when murine calvarial mesenchymal OP9 cells were used to promote differentiation and facilitate the emergence of ECs, approximately 35.7% of cells were CD31 positive after 40 days of co-culture^9^. Thus, a fast and cost-effective method is needed to derive ECs from hPSCs for clinical applications.

According to current knowledge, ECs appear after hematoblast emergence. Many factors, such as *GATA2*, *cFOS*, *ETV6*, *FLI1*, etc., form a network that controls hematopoietic lineage differentiation^10,11^. Friend leukemia virus-induced erythroleukemia-1 (*FLI1*) is a gene of the E-twenty-six (ETS) group that plays an important role in angiogenesis^12^. *FLI1* sits at the top of the transcriptional regulatory hierarchy for hemangioblast specification in vertebrate embryos^13^. By using xenopus and zebrafish embryos, loss of *FLI1* function results in a substantial reduction or absence of hemangioblasts^13^. Hematopoietic lineage differentiation or endothelial specification also requires various kinases and cytokines, such as SCF^14^, vascular endothelial growth factor (VEGF)^15^, PKA^16^, PKC^17^, etc., to activate signaling pathways. The protein kinase C (PKC) signaling pathway has been reported to play a crucial role in the regulation of angiogenesis. PKC-activating phorbol esters were reported to induce angiogenesis^18^. VEGF is a key angiogenesis factor and can be induced by PKC in non-vascular cells. In the classical model, ECs function as targets and effector cells of the PKC–VEGF axis^19^.

Herein, we delineated an easy and quick way to differentiate hESCs into induced endothelial cells (iECs). In this study, simultaneous overexpression of *FLI1* and activation of PKC rapidly and efficiently induced differentiation of hESCs into iECs with a vascular repertoire and morphology-matching endothelial progenitor cells (EPCs) within 3 days without cell sorting. This method represents a new opportunity for understanding and regulating human EC development and may aid in developing interventions for vascular-related diseases.

## Results

### Overexpression of *FLI1* induced hematopoietic lineage differentiation

**Fig. 1:**
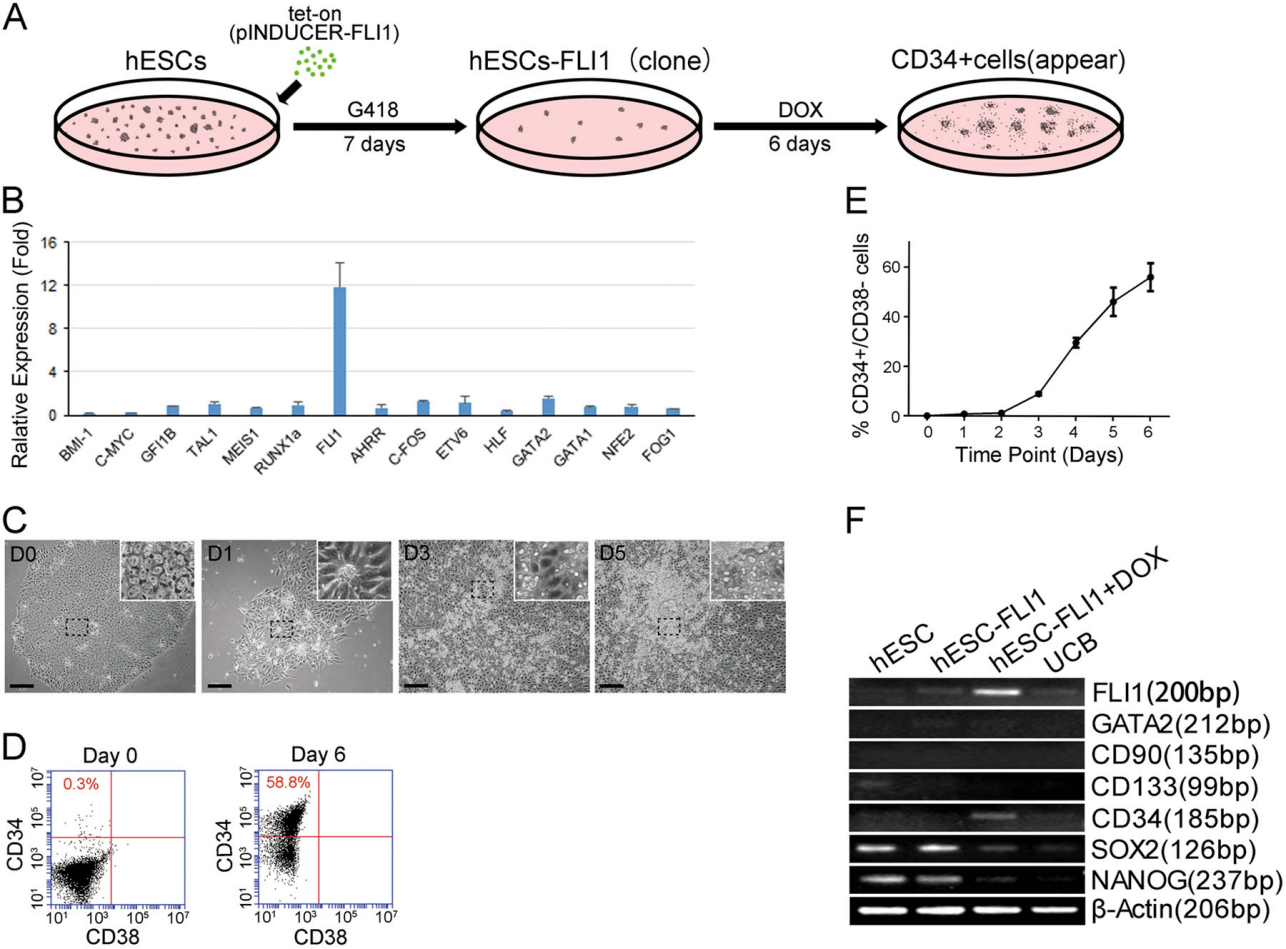
Overexpression of *FLI1* induced hematopoietic differentiation **a** Schematic illustration of transduction into hESCs via *FLI1*. **b** Screening results of CD34 on day 3 after transducing 15 hematopoiesis lineage-relevant genes into hESCs. Columns show the means ± s.d. *n* = 5 independent experiments. **c** Representative images of hESCs-FLI1 after *FLI1* overexpression by DOX on days 0, 1, 3, and 5. Scale bar, 100 μm. **d** Percentage of CD34+/CD38− cells on day 0 (left) and day 6 (right) after *FLI1* overexpression. **e** The percentage of CD34+/CD38− cells gradually increased after *FLI1* overexpression. **f** Expression levels of *FLI1*, *GATA2*, *CD133*, *CD34*, *SOX2* and *NANOG* genes in hESCs, hESCs + FLI1, hESCs + FLI1 + DOX and umbilical cord blood (UCB) cells

Based on the known expression pattern of genes that are critical during hematopoietic lineage differentiation, 15 candidate transcription factors (TFs) were selected for testing their reprogramming ability^10,12,20,21^. We aimed to screen the most optimized TFs for the induction of hemangioblast differentiation from hESCs, and therefore, we established a doxycycline (DOX)-dependent inducible (“Tet-on”) expression system to screen the TFs (*BMI1*, *C-MYC*, *GFI1B*, *TAL1*, *MEIS1*, *RUNX1a*, *FLI1*, *AHRR*, *C-FOS*, *ETV6*, *HLF*, *GATA2*, *GATA1*, *NFE2* and *FOG1*). As CD34 is an important marker gene of hematopoietic lineage, the expression of *CD34* was selected as a marker to evaluate the hematopoietic differentiation degree (Fig. 1a)^22^. On day 2, we found that the expression of CD34 was 12 times that on day 0 only for hESCs overexpressing *FLI1*, while hESCs transduced with other TFs did not exhibit marked changes (Fig. 1b). When *FLI1* was overexpressed by the Tet-on system, the karyoplasmic ratio of hESCs decreased, the shape of the cells became spindle like, and the hESC colonies became loose and gradually falls off (Fig. 1c). Flow cytometric (FCM) analysis revealed that the ratio of CD34+/CD38− increased to 56.2 ± 7% by day 6 (Fig. 1d, e), indicating that *FLI1* induced hematopoietic lineage differentiation. When DOX was added to hESCs-137-FLI1 for 2 days, the RNA expression of *FLI1* and *CD34* (hematopoiesis lineage-related genes) markedly increased, while other hematopoiesis lineage related genes, *CD133* and *GATA2*, were barely detectable, and the expression of the pluripotency related genes *NANOG* and *SOX2* decreased (Fig. 1f). The above results suggested that overexpression of the *FLI1* gene promoted hESCs differentiation toward a hematopoietic lineage.

### PKC activation and *FLI1* overexpression co-stimulate the reprograming of hESCs into ECs

**Fig. 2:**
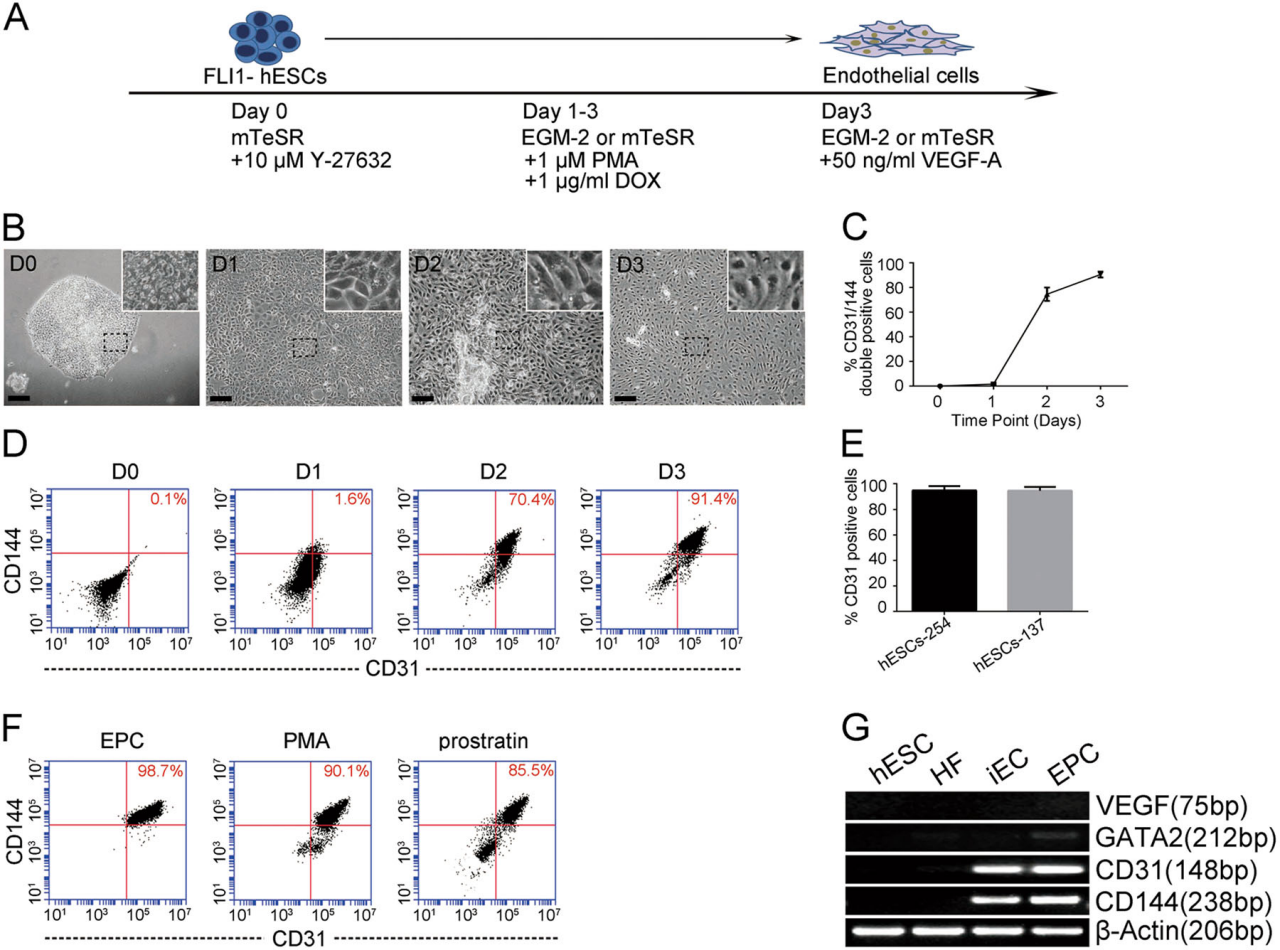
*FLI1* and PKC co-activation mediated hESCs differentiation into iECs **a** Schematic illustration of the EC differentiation strategy from hESCs. **b** Typical morphological images of hESC-EC differentiation on days of 0, 1, 2, and 3. Scale bar, 100 μm. **c** The ratio of CD31+/CD144+ cells gradually increased during induction. Columns represent the mean ± SD; *n* = 5 independent differentiation experiments. **d** Representative results of the percentage of CD31+/CD144+ cells during the induction process detected by FCM. **e** Overexpression of *FLI1* and activation of PKC in different hESC lines (hESC-254 or hESC-137) induced iECs. **f** Overexpressing *FLI1* with different PKC activators (PMA or prostratin) yielded iECs. **g** Expression levels of *VEGF*, *GATA2*, *CD31* and *CD144* genes in hESCs, human fibroblasts (HFs), iECs and EPCs. Columns represent the mean ± SD; *n* = 5 independent differentiation experiments

**Fig. 3:**
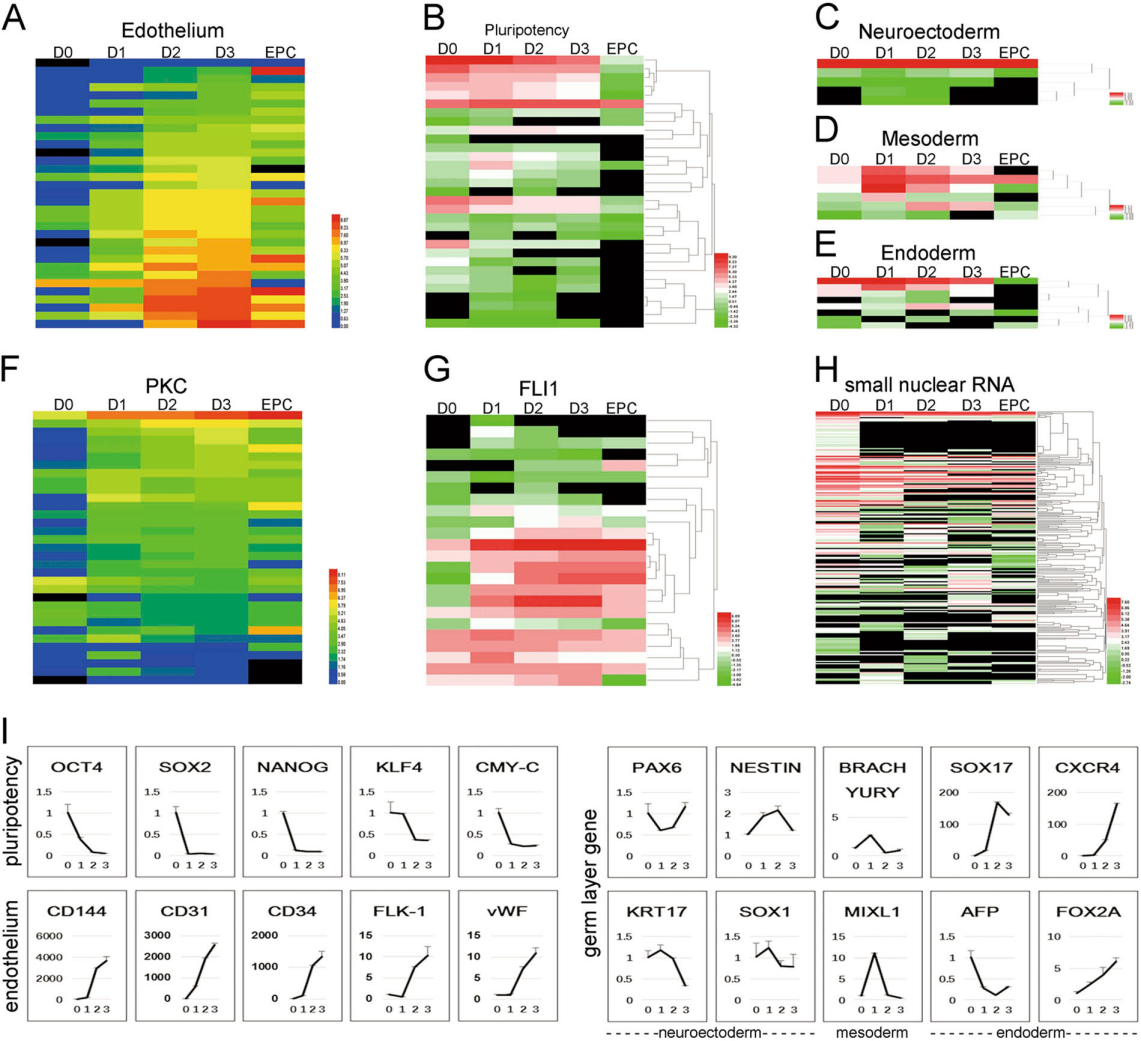
RNA-seq analyses confirmed the vascular cell identity of the iECs Heat map of marker gene panels for endothelium (**a**), pluripotency (**b**), the three germ layers (**c**,** d**,** e**), PKC-related genes (**f** according to KEGG), FLI-related genes (**g** according to Ingenuity Pathway Analysis) and small nuclear RNA (**h**) in hESCs (D0), iECs (D1, D2 and D3) and EPCs. Columns represent genes, and rows are samples. Column *Z*-score transformation was performed on log2 values for each gene with blue denoting a lower and red denoting a higher expression level than the average expression level. Hierarchical clustering of genes and samples are based on average linkage and correlation distance. **i** Dynamic gene expression of representative spatio-temporally regulated genes during hESC-EC differentiation. The mean minimal cycle threshold values were calculated from triplicate reactions

It has been previously reported that activation of PKC is indispensable in the formation of blood vessels^23^. Therefore, we simultaneously overexpressed the *FLI1* gene and activated PKC in hESCs. Interestingly, iECs were obtained within 3 days (Fig. 2a). During induction, the hESCs become loose, undergo deformation and gradually differentiate. Spindle-shaped, triangular and cobblestone-like cells morphologies emerged (Fig. 2b). Upon induction, FCM analysis revealed that the rate of CD144+/CD31+ double-positive cells gradually increased to 89.9 ± 2.7% by day 3 (Fig. 2c, d). The expression of CD144/CD31 was confirmed by quantitative RT-PCR (qRT-PCR; Fig. 3i), which revealed similar results as the FCM analysis. To investigate whether the method was cell line or ES dependent, another ES cell line hESC-254 and two hiPSCs lines (UC013 and SF-iPS) was used under the same protocol. FCM revealed that more than 92 and 87% of hESC-254 cells expressed CD31 (PECAM1) and CD144 (antibody recognize VE-cadherin), respectively (Fig. 2e). CD144+/CD31+ double-positive cells in UC013 and SF-iPS (without hiPSCs-Fli1 monoclonal chosen) have passed 73 and 50%, respectively (Supplementary Fig. 2A). Which indicated that our method can be applied to other ES or hiPSCs cell lines. Similar results were observed when phorbol-12-myristate-13-acetate (PMA) was replaced with another PKC activator, prostratin, to activate PKC. FCM revealed that the rate of CD144+/CD31+ double-positive cells reached 85.5% on day 3 (Fig. 2f), which indicates that PKC activation is important for the induction of ECs. Compared with embryonic stem cells and fibroblasts, iECs and EPCs both highly expressed the endothelial-committed genes *CD144*, *CD31* and *FLK1* and minimally expressed pluripotency genes, as shown in Fig. 2g. Notably, activating PKC or overexpressing *FLI1* alone could not induce iECs. In summary, these data suggest that the combination of PKC activation and *FLI1* overexpression are needed to convert hESCs to ECs.

### iECs exhibit mature EC functional properties

**Fig. 4:**
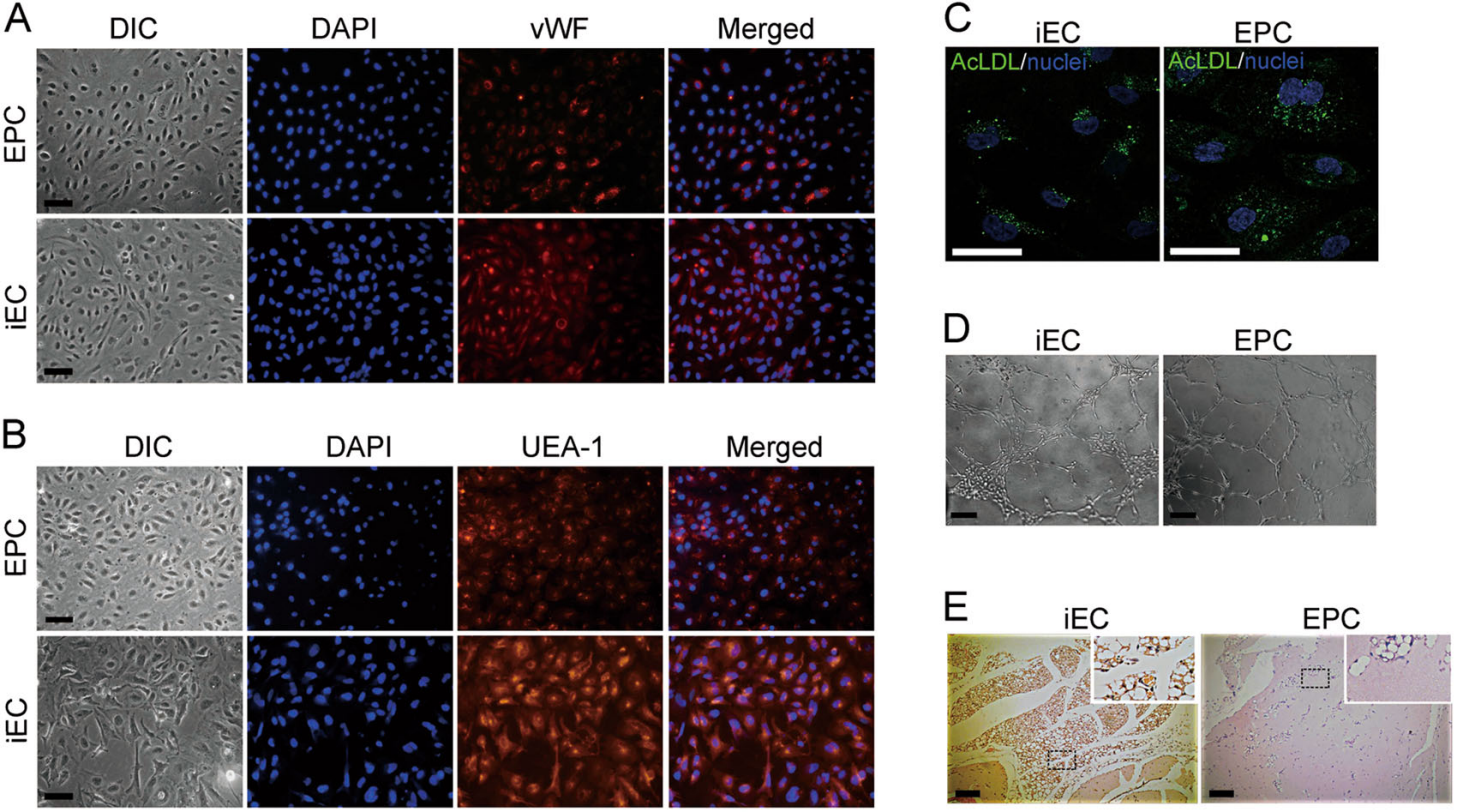
In vitro and in vivo characterization of iECs **a** iECs strongly expressed vWF and bound with UEA-1 **b**. Scale bar, 50 μm. **c** Uptake of fluorescently labeled Ac-LDL by iECs and EPCs. Representative images of three independent experiments. Scale bar, 50 μm. **d** Tubes formed by iECs and EPCs on Matrigel in vitro. Representative image from ten independent experiments. Scale bar, 100 μm. **e** iECs formed vessel-like structures in vivo. Representative images of fibrinogen grafts 14 days after implantation; hematoxylin and eosin staining was conducted for five mice per condition with two implants per mouse (ten implants per condition). Scale bar, 100 μm

We next questioned whether iECs induced via our method possessed true EC functions. Immunofluorescence assays for vWF and ulex europaeus agglutinin 1 (UEA-1) revealed that the iECs and EPCs both exhibited strong induction of multiple endothelial lineage markers, including vWF (Fig. 4a), and a high level of UEA-1 binding (Fig. 4b). Moreover, fluorescently labeled acetylated LDL (Ac-LDL, a characteristic of ECs) was used to estimate the lipid uptake capacity. iECs could efficiently take up Ac-LDL particles (Fig. 4c), which suggested that the induced iECs exhibited mature EC functions. Tube formation assays were performed to assess the angiogenic potential of iECs. There was no difference between the tube formation capacity of the iECs and EPCs according to in vitro tube formation assays, as shown in Fig. 4d. Interestingly, iECs formed more vessel-like structures (90%) than EPCs in the in vivo tube formation assays (10%). Hematoxylin and eosin staining showed that the iECs had more vessel-like structures throughout the implants, and there were numerous red blood cells in the lumen. In contrast, EPCs formed fewer vessels, and no red blood cells were found in the implants (Fig. 4e), which indicated that the iECs were capable of forming blood vessels that were connected to the host vasculature. These findings confirmed that the hESC-derived ECs resembled primary ECs in phenotype as well as functional properties both in vitro and in vivo.

### iECs had a similar transcriptome profile compared to EPCs

To evaluate the transcriptome profile of iECs during induction, RNA-seq was performed for the iECs at four time points (days 0, 1, 2 and 3) during differentiation. The genome-wide analysis results (https://www.ncbi.nlm.nih.gov/geo/query/acc.cgi?acc=GSE103898) of the iECs were then compared to the transcriptomes of EPCs on day 3. The transcriptomes of the iECs differed from those of hESCs (day 0, before induction) and were more similar to those of EPCs (Supplementary Table 1). Upon induction, a significant number of endothelial-committed genes (*CD144*, *CD31*, *vWF*, *FLK-1*, *FLT1*, *KDR* and *CD34*) became robustly up-regulated as time progressed (Fig. 3a). Furthermore, the expression levels of various vascular genes were even high than those in EPCs, which implied that iECs were more similar to authentic ECs. In contrast, the expression of pluripotency gene transcripts (*POU5F1*, *SOX2*, *NANOG*, *UTF1*, and *C-MYC*) decreased rapidly during induction (Fig. 3b), mesodermal genes (*T*, *MIXL1*, and *EOMES*) were only transiently expressed during early stages of iEC differentiation (Fig. 3d), and endoderm and ectoderm genes did not markedly change (Fig. 3c, e). In the VEGF signaling pathway (according to the Kyoto Encyclopedia of Genes and Genomes, http://www.genome.jp/kegg/pathway.html), the expression of almost all PKC-related genes was elevated (Fig. 3f). Approximately 2/3 of *FLI1-*related genes (according to Ingenuity pathway analysis) increased, while the rest did not significantly change (Fig. 3g). Almost all of the small RNAs that regulate cell development, physiology and pathogenesis were downregulated (Fig. 3h)^24^. The expression of endothelial fate-related RNA was detected by qRT-PCR, which revealed the same results as the RNA-seq analysis (Fig. 3i). The above results indicate that the key signaling pathway for differentiating hESCs into iECs resembled their primary counterpart EPCs and may still have followed the normal endothelial specification process except that differentiation into iECs occurred more sharply.

### PKC is essential for hESCs differentiation into ECs

**Fig. 5:**
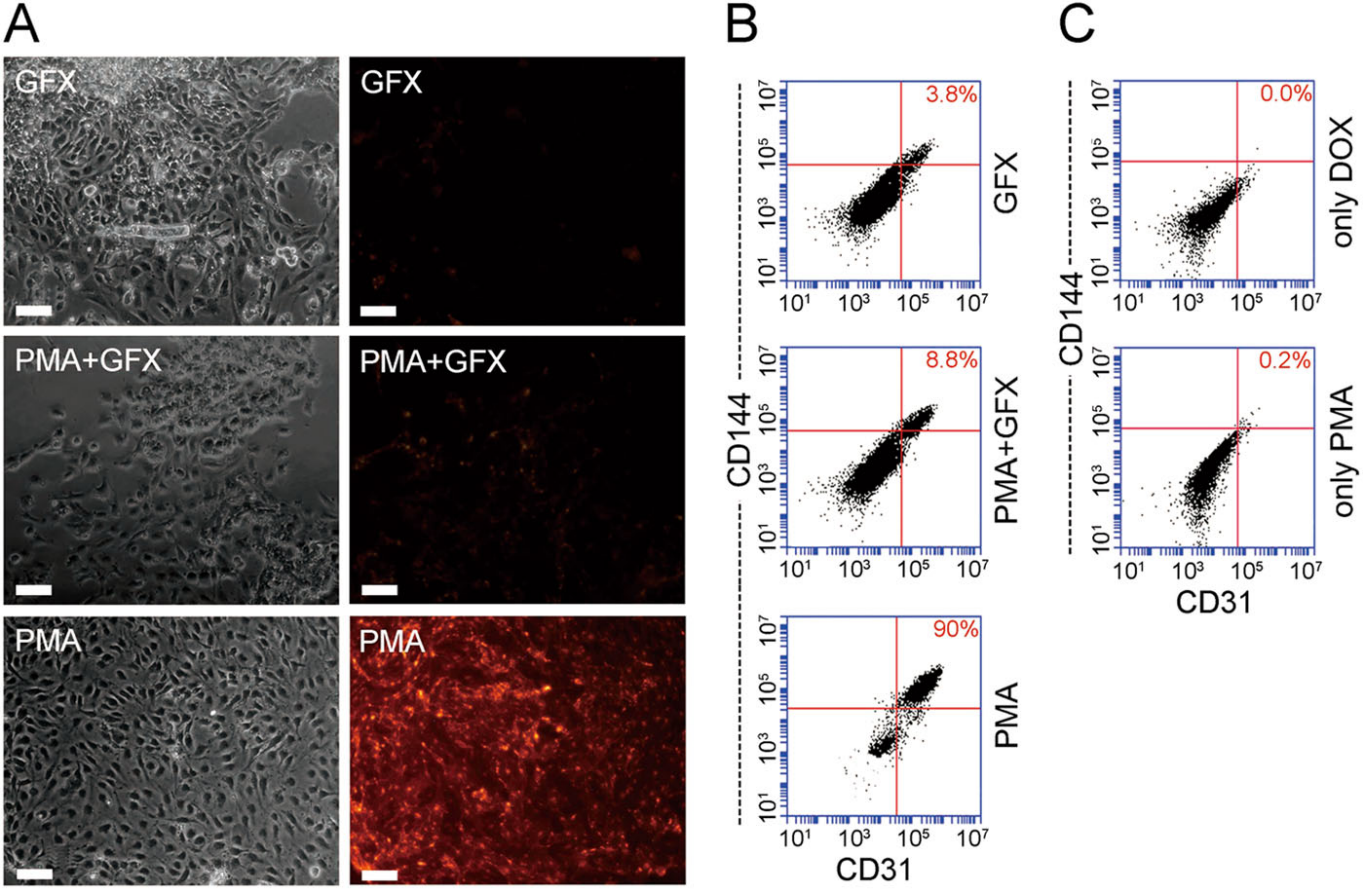
PKC inhibitor inhibits the generation of iECs **a** GFX partially inhibits the generation of iECs from hESCs detected by UEA-1 binding assays. Scale bar, 50 μm. **b** GFX nearly inhibited the production of CD31+/CD144+ cells as detected by flow cytometric. **c** The addition of only DOX or PMA did not induce CD31+/CD144+ cells

To emphasize the role of PKC, we inhibited PKC with GF109203X (GFX) at the beginning of the induction process. After induction, many cells fallen off (10%), while more cells remained in the undifferentiated state (87.5%) (Fig. 5a). Immunofluorescence analysis showed that few cells bound with UEA-1 (Fig. 5a). FCM detection showed that the rate of CD144+/CD31+ double-positive cells in the GFX + PMA group was only 8.8%, which was significantly lower than the 85.5% observed in the PMA group and approached the 3.8% observed for the negative group (Fig. 5b). Activating PKC or overexpressing *FLI1* alone failed to induce the generation of iECs (Fig. 5c). These results suggested that PKC activation in our system indeed plays an essential role in the determination of endothelial lineage.

## Discussion

ECs have great therapeutic potential for treating vascular disorder diseases. However, purification, isolation and expansion of adult sources have been reported to be technically cumbersome^12^. Here, we report a fast, highly efficient and cost-effective method to induce differentiation of hESCs into iECs. Simultaneously overexpressing the *FLI1* gene and activating PKC can reprogram hESCs into iECs within 3 days without cell sorting or magnetic affinity separation. The generated iECs possess the same morphology and angiogenic repertoire as ECs. In vivo tube formation assays revealed that the iEC group formed more vessels than the EPC group, and numerous red blood cells were found in the lumen, which implied that the vessels could connect to the host vasculature. According to the RNA-seq data, the expression of venous endothelial marker *NRP2*, artery endothelial marker *NOTCH1* (*Jagged1*) and smooth muscle cell marker *TGFB1* (*a-SMA*) were all markedly induced and reached a higher level on day 3 (Fig. 3a). Our iECs appear similar to early stage ECs and have a better vasculogenic capacity than ECs. The induction process of ECs based on our protocol was more efficient and faster than previously reported protocols using cytokines^25,26^. RNA-seq analyses showed that the iECs were similar to adult ECs in which vascular-specific genes were activated and non-vascular genes were silenced, suggesting that once cells have been sufficiently epigenetically activated, they may develop toward EC lineages and at least partly follow the “natural” development pathway.

The *FLI1* gene and PKC have been reported to be responsible for the induction of EC fate^13,17^. We found that activating PKC or overexpressing *FLI1* alone in our induction system failed to induce the generation of iECs. Hematopoietic signaling pathway is a complicated process, and the key factors remain unknown. In this research, we found that only overexpressing the *FLI1* gene produced the largest number of hematopoietic lineage marker gene CD34+ cells, but CD34+ cells could not form colonies. *FLI1* is an *ETS* TF and plays key roles in the hematopoietic signaling pathway. RNA-seq data showed that the expression of hematopoietic lineage-related genes, such as *ERG*, *GATA2* and *MEIS1* were all up-regulated after activating PKC and overexpressing *FLI1*, which suggested that *FLI1* sat at the top of the hematopoietic differentiation hierarchy and may govern endothelial fate from hESCs.

PKC signaling pathway is a classical pathway involved in many biological processes including endothelial development. Many studies have reported that activating PKC could generate VEGF^19,27^. Our study revealed that co-activating *FLI1* and PKC promoted EC differentiation from hESCs, while activating PKC or overexpressing *FLI1* alone could not induce iECs. VEGF is a crucial regulator of endothelial differentiation. We speculated that activating the PKC signaling pathway in the hematopoietic lineage could drive EC differentiation through VEGF. Activation of PKC alone could up-regulate the expression of VEGF, while overexpressing *FLI1* alone could not. RNA-seq analysis revealed that, after co-activating PKC and *FLI1*, VEGF was robustly increased on day 1 and then decreased rapidly. PKC inhibitor could inhibit hESC-iEC differentiation in VEGF-containing EGM medium, while in mTeSR (No. 05850, STEMCELL) medium excluding VEGF, iECs could be obtained from hESCs. These results illustrate that the PKC signaling pathway may play a more important role than VEGF in the generation of ECs from hESCs.

In a recent report, transient Etv2 expression of constitutive *Erg1* and *FLI1* co-expression induced direct reprogramming of mature amniotic cells into ECs^12^. PKCα-mediated non-canonical signaling pathways are required for the differentiation of mouse embryonic stem cells into ECs. We hypothesized that overexpressing *FLI1* drives hESCs toward a hematopoietic lineage and that activation of PKC activates the VEGF signaling pathway. Both function together to differentiate hESCs into iECs, although an interesting phenomenon to be elucidated is how iECs were generated in such a short time.

Recently, Christoph Patsch and Chad A. Cowan’s group reported that, after specific recombinant growth factors were added to culture media and used in two-dimensional culture, approximately 61.88–88.8% cells were CD144+ after 6 days of induction^26^. Ping Zhou’s group derived 94–97% CD31+ and 78–83% CD144+ cells from hiPSCs and hESCs in 8 days^25^. Both groups produced a large amount of ECs in a relatively short time, but both methods required cytokines, and the internal mechanism of EC fate remains unclear. Our protocol has several advantages compared with other methods used to induce vascular cells from hESCs. To our knowledge, 3 days are the shortest amount of time in which functional ECs have been generated from hESCs. In addition, our method is economical and does not require expensive cytokines, as only simple chemical molecules are needed. Our method is easy to reproduce, does not require tedious procedures, and can produce high quality cells to enable high-throughput screening and large-scale analyses. It is an effective method, the iECs are uniform, and more than 92 and 87% of cells were CD31+ and CD144+, respectively, indicating nearly pure populations of ECs.

However, the iECs generated here incorporated genetic modifications and are far from clinical applications. Nonetheless, this robust method is likely to serve as a new way to engineer artificial blood vessels and tissues for clinical and scientific research. More importantly, the detailed molecular mechanism of this in vitro direction differentiation of ECs from hESCs may help to better elucidate angiogenesis and control endothelial fate. Overall, our findings provide new insight into the regulatory signaling network of EC development.

## Materials and methods

### hPSCs lines derivation and cell culture

hESC cell lines hESC-137^28^, and hESC-254 used in this research were established in our laboratory and were derived from surplus embryos from IVF treatment with the informed consent of the patients. Another two hiPSCs lines UC013^29^, and SF-iPS^30^ were purchased from Guangzhou Institutes of Biomedicine and Health, Chinese Academy of Sciences (Supplementary Fig. 1). This experiment was approved and guided by the ethical committee of Reproductive and Genetic Hospital of CITIC-Xiangya. All cell lines were routinely tested for mycoplasma contamination and were negative throughout this study. All hESC lines were plated on MEF feeders. The cells were cultured in Dulbecco’s modified Eagle’s medium/F-12 supplemented with 15% knockout serum replacement, 2 mM nonessential amino acids, 2 mM l-glutamine, 0.1 mM β-mercaptoethanol and 4 ng/ml human recombinant basic fibroblast (bFGF) (all from Invitrogen). The medium was changed every day until the embryo outgrowth was observed. Then the outgrowth was transmitted onto fresh MEF feeder layers and obtained the hESCs-colony morphology during prolonged culture. hESCs colonies were mechanically passaged every 7 days.

### Construction of hESCs-FLI1 condithonal expression cell lines

We use pINDUCER20 plasmid (Supplementary Fig. 2B) to construct tet-on regulation system that overexpress *FLI1* gene. 4.5 μg of lentiviral vector (*FLI1*) and 3 μg of PCMV and 1.5 μg VSV-G were contransfected in 293FT cells by using lipofectamine 2000. Supernatants were collected 48 h after transfection. Supernatants containing infectious particles were harvested 48 h after transfection and then were concentrated by ultracentrifugation. After filtering through a 0.45-μm filter, viral supernatants were used to transduce undifferentiated hESC-137, hESC-254, UC01321, and SF-iPS. After two passages, hESCs were cultured for 2 weeks, then screened using G418 (11811023, Gibco). To get purified hESCs-FLI1, another screen of G418 was used after expansion and monoclonal were chosen to culture (UC01321, and SF-iPS didn’t go through this operation).

### Endothelial differentiation protocols

For differentiation, hESCs-FLI1 were mechanically isolated and then plated on matrigel (354234, Corning)-coated plates in mTeSR1 (05850, STEMCELL) with 10 µM ROCK inhibitor Y-27632 (688002, Calbiochem). After 24 h, the medium was changed with mTeSR1 or EGM-2 (CC-3162, Lonza) supplemented with 1 µM PMA (sc-3576, Santa Cruz) and 1 µg ml^−1^ DOX (D9891, Sigma-Aldrich). After 3 days, supplements were changed with 50 ng ml^−1^ VEGF-A (96-100-20-10, PEPROTECH). The medium was replaced every day.

### FCM analysis

For FCM analysis, all FACS antibodies were directly labeled and obtained from BD Pharmingen (CD34-PE, CD38-FITC, CD144-PE, CD31-FITC, IgG1-PE, and IgG1-FITC). All antibodies (5 μl) were incubated in 50-μl-reaction volume at 37 °C for 30 min before detection. An isotype-matched antibody was served as a negative control. Cell analysis and sorting were performed on Accuri C6 (BD Biosciences). Data were analyzed using BD Accuri C6 software.

### Quantitative RT-PCR (qRT-PCR)

Total RNA was isolated with Trizol (15596018, Life Technologies) and CDNA was synthesized by Transcriptor First Strand CDNA Synthesis kit (04897030001, Roche) according to the manufacturer’s instructions. Quantitative PCR was performed on a LightCycler 480IIPCR System (Roche) using SYBR Green PCR mix (04887352001, Roche). RT-PCR was also done using GoTaq Green Master Mix (A6001, Promega). Human-specific primer pairs for tested were supplied in Supplementary Table 2. Relative expression was quantified using the comparative cycle threshold method, threshold cycles were normalized to β-Actin. Transcript amplification was analyzed by 1.5% agarose gel electrophoresis of the qPCR or RT-PCR products. Each set of reactions was repeated using cDNA from at least three independent experiments.

### Immunofluorescence staining

Cultured cells were washed once with Dulbecco's phosphate-buffered saline (DPBS; 8115028, Gibco) and fixed with ice-cold 4% paraformaldehyde (P6148, Sigma) for 10 min at 37 °C. Then treated by mixed solution of 0.5% TritonX -100 (T9284, Sigma) and 10% donkey serum (017-000-121, Jackson Immuno Reserach) for 30 min at room temperature for permeabilizing and blocking. The cells were then incubated with primary antibodies against vWF (1:50, 555849, BD) overnight in the dark at 4 °C. After three washes with DPBS, cells were incubated in 1% BSA (E661003, Sangon) in PBS containing secondary antibodies Alexa Fluor^®^ 555 (1:1000, A32727, Invitrogen). Nuclei were counterstained with DAPI (1:1000, D9564, Sigma). Images were acquired in an epifluorescence microscopy (Nikon Eclipse TE2000-U).

### UEA-1 binding

UEA-1 (1:400, FL-1061, Vector Labs) was added to the medium and incubated in the dark at 37 °C for 30 min. Thereafter, cells were washed once with DPBS and fixed with 4% PFA (paraformaldehyde, PFA) for 10 min. Nuclei were counterstained with DAPI. Images were acquired in an epifluorescence microscopy.

### LDL uptake assay

Alexa Fluor 488 acetylated low-density lipoprotein (L-23380, DiI-ac-LDL; Molecular Probes/Life Technologies) was diluted to 10 μg/ml in complete growth medium and added to the cells; the mixture was allowed to incubate at 37 °C for 4 h. Thereafter, cells were washed once with DPBS and fixed with 4% PFA for 10 min. DNA was counterstained with DAPI. Finally visualized with a confocal laser scanning microscope (Olympus IX81 and (CLSM) Olympus FV1000, Tokyo, Japan).

### In vivo vasculogenic assay

Animal procedures were approved by the Ethics Committee of Central South University, Changsha, China. Ten males (five per condition) 6-week-old male BALB/c nude mice were purchased from SLAC Co., Ltd (Shanghai, China). iEC and EPC (1.2 × 10^6^ cells) were resuspended in 200 μl matrigel and then implanted subcutaneously into the dorsal flank of the male BALB/c nude mice. After 14 days, implants were harvested for tissue analysis. Then implants were fixed overnight in 4% paraformaldehyde and stain with haematoxylin and eosin (H&E). The structures were photographed under an inverted microscope (leica).

### In vitro vasculogenic assay

Firstly, matrigel were seeded on 96-well plates (50 μl/well). After concretion, (3–5) × 10^4^ cells (EPC or iEC) were resuspended in 300 μl endothelial differentiation medium and placed on 96-well plates. Then plates were incubated at 37 °C and 5% CO_2_ for 4 h. The structures were photographed under phase contrast microscope (TE2000-U, Nikon).

### RNA-seq and analysis

Total RNA samples were extracted with TRIzol reagent and treated with DNase I to degrade any possible DNA contamination. Then the mRNA is isolated by using the oligo (dT) magnetic beads and mixed with fragmentation buffer. Then the first strand of cDNA is synthesized by using reverse transcriptase and random hexamer primers. The cDNA is purified with magnetic beads. End reparation and 3ʹ-end single nucleotide A (adenine) addition is then performed. Finally, sequencing adapters and PCR are ligated to the fragments. The products are ready for sequencing via Illumina HiSeqTM 2000 after sample library was established. Primary sequencing data are filtered into clean reads. After passing quality control, we will proceed with downstream analysis including gene expression and deep analysis based on gene expression.

### Statistical analysis

Unless otherwise cited, all data are expressed as the mean ± SD, and were analyzed using SPSS 19.0 software (SPSS, Chicago, IL, USA). Intergroup differences were explored using one-way analysis of variance and the least significant difference post hoc tests. *P*-value < 0.05 was regarded as significant. All experiments were repeated at least twice, and data from one representative experiment were shown.

## Electronic supplementary material

hESC and hiPSC cell lines expressed pluripotent markers

FLI1 and PKC co-activation mediated hiPSCs differentiation into iECs

The transcriptomes of the iECs

primers sequences

Supplementary Figure Legends
