# Supplementary figures and images for: FLI1 and PKC co-activation promote highly efficient differentiation of human embryonic stem cells into endothelial-like cells
Source: Cell Death Dis. 2018 Jan 26;9(2):131. doi: 10.1038/s41419-017-0162-9 (PMC5833666; doi:10.1038/s41419-017-0162-9)

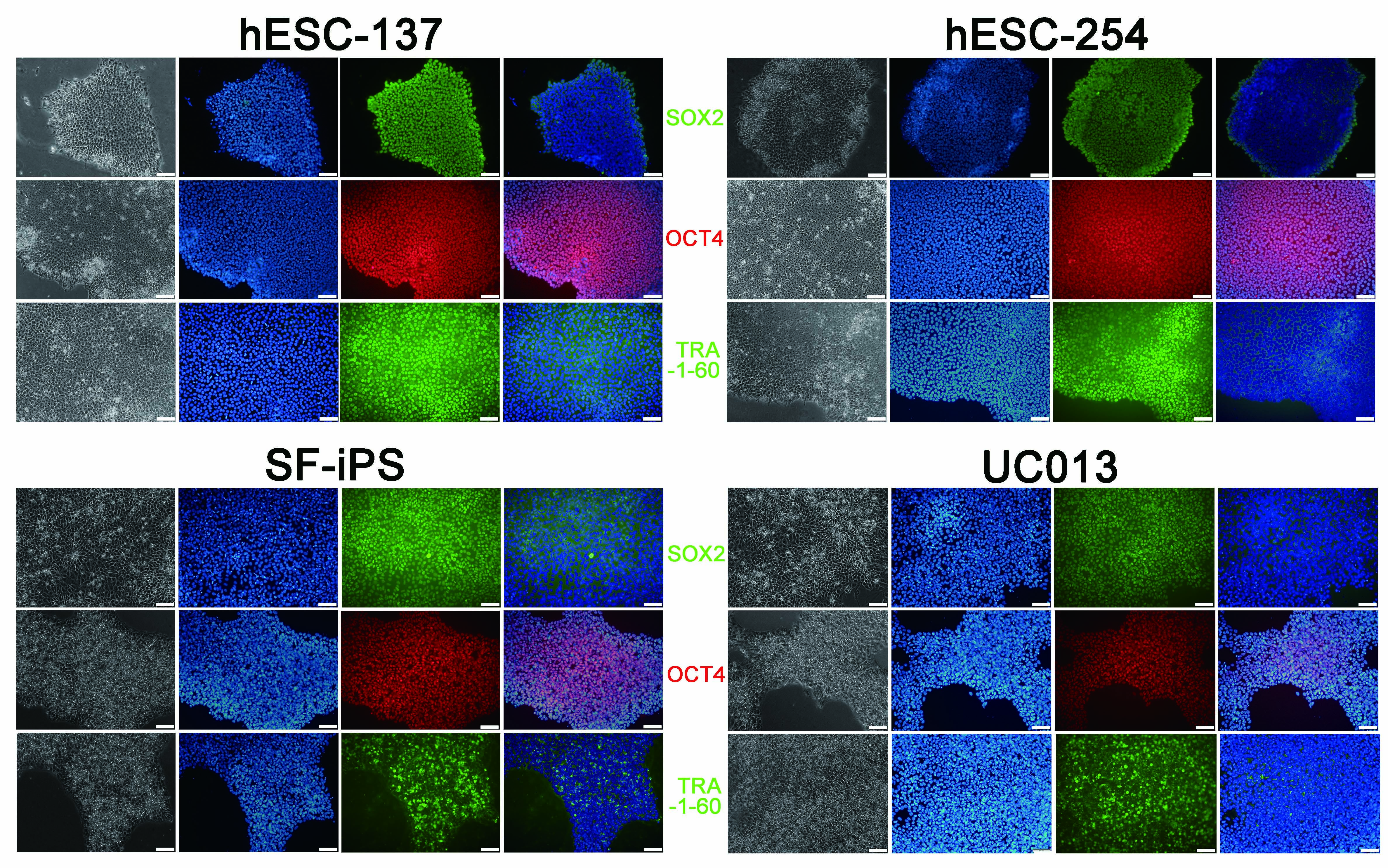

Supplement: Supplementary file 1 — hESC and hiPSC cell lines expressed pluripotent markers [file 41419_2017_162_MOESM1_ESM.jpg]

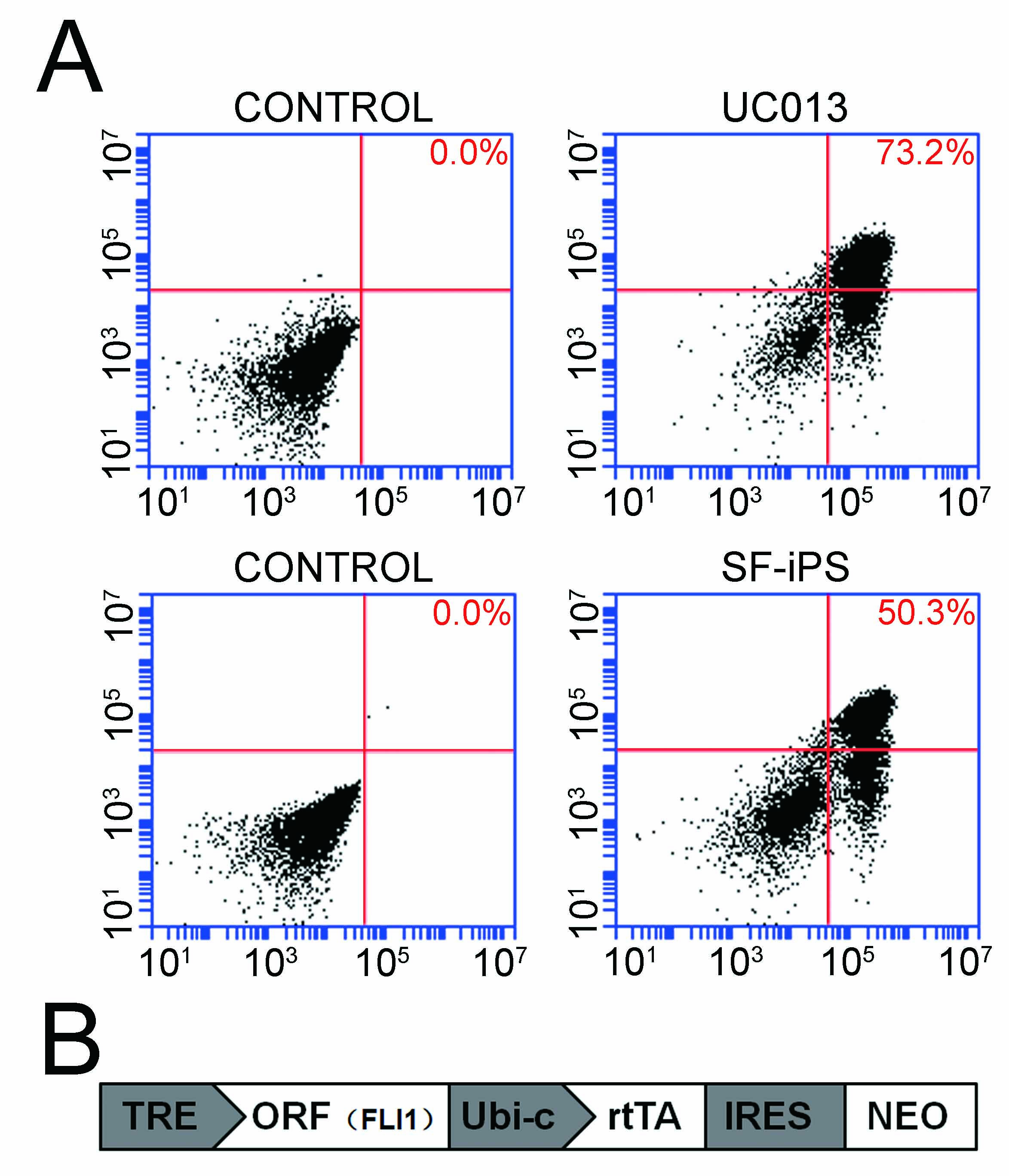

Supplement: Supplementary file 2 — FLI1 and PKC co-activation mediated hiPSCs differentiation into iECs [file 41419_2017_162_MOESM2_ESM.jpg]
