# Supplementary material for: FLI1 and PKC co-activation promote highly efficient differentiation of human embryonic stem cells into endothelial-like cells
Source: Cell Death Dis. 2018 Jan 26;9(2):131. doi: 10.1038/s41419-017-0162-9 (PMC5833666; doi:10.1038/s41419-017-0162-9)
Supplement: Supplementary file 3 — The transcriptomes of the iECs [file 41419_2017_162_MOESM3_ESM.docx]

| The transcriptomes of the iECs | | | | | |  |
| --- | --- | --- | --- | --- | --- | --- |
|  |  |  |  |  |  |  |
| **A Endothelium** | | | | | |  |
| symbol | D0 | D1 | D2 | D3 | EPC |  |
| GPR146 | 0 | 1.45 | 1.46 | 1.24 | 2.18 |  |
| VWF | 0.22 | 0.35 | 3.72 | 6.18 | 631.52 |  |
| CXCR7 | 1.38 | 0.96 | 4.11 | 11.51 | 2.91 |  |
| NOS3 | 1.27 | 39.47 | 26.5 | 17.07 | 24.17 |  |
| ANGPT2 | 0.8 | 1.64 | 3.38 | 17.23 | 34.15 |  |
| GATA2 | 0.52 | 12.82 | 15.96 | 17.59 | 9.82 |  |
| PTPRB | 0.27 | 1.74 | 13.56 | 20.33 | 36.84 |  |
| HERPUD1 | 11.01 | 41.57 | 26.03 | 25.18 | 32.13 |  |
| S1PR1 | 0.42 | 2.64 | 16.9 | 35.1 | 68.29 |  |
| TEK | 3.98 | 8.23 | 35.49 | 35.15 | 33.77 |  |
| GPR4 | 0.41 | 9.94 | 34.25 | 38.95 | 19.86 |  |
| GPR116 | 0 | 2.58 | 34.74 | 45.39 | 34.54 |  |
| FLT4 | 1.91 | 22.21 | 62.94 | 56.21 | 9.89 |  |
| HEY2 | 3.2 | 4.11 | 42.5 | 60.4 | 0 |  |
| NRP2 | 9.45 | 41.68 | 83.24 | 66 | 83.5 |  |
| HEY1 | 0.79 | 1.27 | 47.43 | 67.85 | 1.36 |  |
| RASIP1 | 1.86 | 44.89 | 83.74 | 89.54 | 34.96 |  |
| TIE1 | 0.55 | 42.53 | 102.44 | 99.57 | 223.91 |  |
| NOTCH1 | 14.32 | 49.12 | 103.93 | 108.41 | 28.45 |  |
| NOTCH1 | 14.32 | 49.12 | 103.93 | 108.41 | 28.45 |  |
| JAG1 | 8.02 | 45.03 | 87.05 | 115.58 | 11.72 |  |
| SOX7 | 0.97 | 59.03 | 153.01 | 121.05 | 24.3 |  |
| DLL4 | 0 | 15.32 | 73.9 | 152.95 | 9.18 |  |
| CD34 | 0.07 | 10.19 | 143.12 | 168.84 | 34.75 |  |
| PECAM1 | 0.64 | 26.89 | 117.09 | 169.78 | 413.12 |  |
| TGFB1 | 6.91 | 101.1 | 210.39 | 175.12 | 127.78 |  |
| EFNB2 | 8.01 | 15.42 | 88.69 | 198.52 | 15.1 |  |
| THY1 | 144.38 | 171.33 | 230.3 | 251.43 | 0.15 |  |
| CDH5 | 0.09 | 15.42 | 231.88 | 357.09 | 558.25 |  |
| KDR | 24.19 | 17.78 | 306.82 | 408.58 | 108.35 |  |
| ESAM | 0.29 | 140.3 | 378.52 | 439.41 | 198.1 |  |
| FLT1 | 28.78 | 54.47 | 229.74 | 454.74 | 85.12 |  |
| ESM1 | 0.19 | 0.88 | 156.64 | 723.56 | 351.07 |  |
|  |  |  |  |  |  |  |
|  |  |  |  |  |  |  |
| **B Pluripotency** | | | | | |  |
| Symbol | D0 | D1 | D2 | D3 | EPC |  |
| POU5F1 | 1146.38 | 613.85 | 178.16 | 123.05 | 4.19 |  |
| DPPA4 | 187.63 | 53.44 | 42.5 | 49.65 | 0.95 |  |
| TUBB3 | 138.05 | 201.44 | 195.76 | 201.17 | 133.93 |  |
| ZFP42 | 119.11 | 47.17 | 20.57 | 17.56 | 1.19 |  |
| SOX2 | 72.9 | 2.91 | 4.97 | 5.06 | 0 |  |
| NANOG | 70.1 | 12.43 | 13 | 10.87 | 1.47 |  |
| SALL4 | 61.17 | 30.4 | 22.61 | 18.3 | 0.3 |  |
| NR6A1 | 38.31 | 31.28 | 14.99 | 8.7 | 0.28 |  |
| ST14 | 19.57 | 25.52 | 12.69 | 7.62 | 0.05 |  |
| FOXD3 | 7.21 | 0.35 | 0.53 | 0.79 | 0 |  |
| DPPA2 | 4.89 | 2.66 | 0 | 0 | 0 |  |
| EOMES | 3.23 | 16.53 | 6.82 | 2.78 | 0 |  |
| UTF1 | 3.2 | 0.97 | 0.16 | 0.48 | 0 |  |
| SNAI1 | 2.87 | 17.46 | 13.27 | 7.68 | 8.91 |  |
| DPPA5 | 2.59 | 0.87 | 0 | 0.29 | 0 |  |
| MIXL1 | 2.17 | 39.5 | 5.21 | 2.17 | 0.26 |  |
| RIPK4 | 2 | 9.79 | 2.27 | 1.07 | 0 |  |
| AFP | 1.98 | 0.9 | 0.18 | 0.99 | 0 |  |
| DPPA3 | 1.79 | 0.54 | 1.63 | 0.54 | 0.34 |  |
| PAX6 | 0.98 | 0.32 | 0.66 | 1.05 | 0.08 |  |
| GATA4 | 0.96 | 0.91 | 2.42 | 1.6 | 0 |  |
| T | 0.9 | 3.37 | 0.66 | 0.9 | 0 |  |
| SNAI2 | 0.52 | 0.87 | 4.99 | 3.73 | 0.42 |  |
| KHDC3L | 0.36 | 0 | 0 | 0 | 0 |  |
| TMPRSS4 | 0.16 | 0 | 0.32 | 0 | 0 |  |
| SHH | 0.12 | 0.7 | 0 | 0 | 0.56 |  |
| ZIC1 | 0.1 | 0.07 | 0.07 | 0.1 | 0 |  |
| SOX1 | 0 | 0.31 | 0.09 | 0 | 0 |  |
| NKX6-1 | 0 | 0.17 | 0.17 | 0 | 0 |  |
| FOXA1 | 0 | 0.48 | 0 | 0.32 | 0.05 |  |
| PDX1 | 0 | 0.57 | 0.22 | 0 | 0 |  |
|  |  |  |  |  |  |  |
|  |  |  |  |  |  |  |
| **C Neuroectoderm** | | | | | |  |
| symbol | D0 | D1 | D2 | D3 | EPC |  |
| TUBB3 | 138.05 | 201.44 | 195.76 | 201.17 | 133.93 |  |
| PAX6 | 0.98 | 0.32 | 0.66 | 1.05 | 0.08 |  |
| ZIC1 | 0.1 | 0.07 | 0.07 | 0.1 | 0 |  |
| SOX1 | 0 | 0.31 | 0.09 | 0 | 0 |  |
| NKX6-1 | 0 | 0.17 | 0.17 | 0 | 0 |  |
|  |  |  |  |  |  |  |
|  |  |  |  |  |  |  |
| **D Mesoderm** | | | | | |  |
| symbol | D0 | D1 | D2 | D3 | EPC |  |
| EOMES | 3.23 | 16.53 | 6.82 | 2.78 | 0 |  |
| SNAI1 | 2.87 | 17.46 | 13.27 | 7.68 | 8.91 |  |
| MIXL1 | 2.17 | 39.5 | 5.21 | 2.17 | 0.26 |  |
| T | 0.9 | 3.37 | 0.66 | 0.9 | 0 |  |
| SNAI2 | 0.52 | 0.87 | 4.99 | 3.73 | 0.42 |  |
| HOXA1 | 0.08 | 0.47 | 0.22 | 0 | 0.96 |  |
|  |  |  |  |  |  |  |
|  |  |  |  |  |  |  |
| **E Endoderm** | | | | | |  |
| symbol | D0 | D1 | D2 | D3 | EPC |  |
| ST14 | 19.57 | 25.52 | 12.69 | 7.62 | 0.05 |  |
| RIPK4 | 2 | 9.79 | 2.27 | 1.07 | 0 |  |
| AFP | 1.98 | 0.9 | 0.18 | 0.99 | 0 |  |
| GATA4 | 0.96 | 0.91 | 2.42 | 1.6 | 0 |  |
| TMPRSS4 | 0.16 | 0 | 0.32 | 0 | 0 |  |
| SHH | 0.12 | 0.7 | 0 | 0 | 0.56 |  |
| FOXA1 | 0 | 0.48 | 0 | 0.32 | 0.05 |  |
| PDX1 | 0 | 0.57 | 0.22 | 0 | 0 |  |
|  |  |  |  |  |  |  |
|  |  |  |  |  |  |  |
| **F PKC** | | | | | |  |
| Symbol | D0 | D1 | D2 | D3 | EPC |  |
| SH3BGRL3 | 54.58 | 149.75 | 180.87 | 217.37 | 411.09 |  |
| LIMS1 | 11.9 | 31.3 | 59.75 | 78.3 | 46.81 |  |
| MAPK11 | 0.38 | 23.33 | 33.57 | 53.81 | 14.85 |  |
| YPEL2 | 1 | 10.93 | 23.86 | 36.94 | 15.19 |  |
| ARHGAP29 | 1.7 | 15.82 | 19.56 | 33.76 | 58.54 |  |
| LIMS3L | 1.8 | 6.11 | 18.35 | 29.06 | 20.59 |  |
| TGFB1I1 | 2.53 | 6.1 | 16.54 | 28.45 | 36.69 |  |
| SRC | 13.79 | 30.5 | 33.88 | 26.03 | 14.3 |  |
| MAPK12 | 7.32 | 24.98 | 13.68 | 18.38 | 19.92 |  |
| MAPK12 | 7.32 | 24.98 | 13.68 | 18.38 | 19.92 |  |
| NOS3 | 1.27 | 39.47 | 26.5 | 17.07 | 24.17 |  |
| SPHK1 | 0.5 | 12.17 | 9.06 | 11.49 | 67.04 |  |
| PIK3CA | 4.09 | 9.11 | 8.93 | 9.76 | 11.61 |  |
| SHC2 | 1.81 | 9.27 | 7.87 | 9.38 | 2.93 |  |
| PIK3R1 | 2.77 | 10.17 | 6.98 | 9.23 | 6.36 |  |
| PPP3CA | 6.46 | 13.01 | 10.83 | 9.17 | 11.29 |  |
| CPNE8 | 2.35 | 4.83 | 7.67 | 8.21 | 4.47 |  |
| PTGS2 | 0.24 | 3.47 | 5.82 | 8.01 | 6.26 |  |
| LOXL1-AS1 | 2.52 | 6.29 | 8.73 | 7.66 | 0.4 |  |
| PPP3CC | 2.14 | 6.05 | 7.12 | 6.58 | 13.86 |  |
| RAC3 | 53.11 | 23.07 | 9.96 | 5.36 | 21.07 |  |
| MAPKAPK3 | 32.67 | 9.81 | 5.21 | 5.29 | 9.51 |  |
| CPNE5 | 0 | 0.98 | 3.52 | 4.74 | 1.83 |  |
| PIK3CB | 8.04 | 5.02 | 4.81 | 4.67 | 5.45 |  |
| HMHA1 | 11.43 | 5.24 | 4.18 | 4.6 | 6.73 |  |
| LRRC45 | 8.96 | 2.57 | 3.35 | 4.22 | 5.51 |  |
| RAC2 | 1.21 | 7.2 | 7.74 | 3.53 | 110.6 |  |
| VEGFA | 6.66 | 36.05 | 4.7 | 3 | 2.8 |  |
| NFATC2 | 0.37 | 1.34 | 1.35 | 2.14 | 6.21 |  |
| CHN2 | 1.16 | 10.85 | 2.03 | 1.36 | 0.91 |  |
| PIK3R6 | 0.18 | 1.91 | 1.8 | 0.36 | 0 |  |
| PRKCG | 2.14 | 6.74 | 2.46 | 0.35 | 0 |  |
| PLEK | 0 | 1.34 | 0.19 | 0.25 | 0 |  |
|  |  |  |  |  |  |  |
|  |  |  |  |  |  |  |
| **G FLI1** | | | | | |  |
| symbol | D0 | D1 | D2 | D3 | EPC |  |
| GATA1 | 0 | 0.12 | 0 | 0 | 0 |  |
| GFI1B | 0 | 3.26 | 0.34 | 0 | 0 |  |
| PF4 | 0.21 | 0 | 0.43 | 0 | 0 |  |
| CSF1R | 0.36 | 0.18 | 0.18 | 0.09 | 0 |  |
| IL2RA | 0.43 | 0.11 | 0.29 | 0.25 | 0.35 |  |
| IKZF1 | 0.15 | 0.71 | 0.89 | 0.37 | 0 |  |
| HOXB4 | 0 | 0 | 0.45 | 0.45 | 9.28 |  |
| IL6 | 0 | 1.09 | 0.31 | 0.46 | 1.04 |  |
| GP1BA | 0.66 | 3.74 | 1.25 | 0.88 | 0.21 |  |
| ZFPM1 | 0.95 | 4.23 | 3.35 | 1.8 | 2.69 |  |
| VEGFA | 6.66 | 36.05 | 4.7 | 3 | 2.8 |  |
| MEIS1 | 0.34 | 0.23 | 3.8 | 6.2 | 1.2 |  |
| RUNX1 | 0.6 | 3.77 | 8.05 | 8.17 | 6.69 |  |
| ELK1 | 13.55 | 14.07 | 13.46 | 10.49 | 6.99 |  |
| SP3 | 21.12 | 17.6 | 13.2 | 12.14 | 9.11 |  |
| RARA | 4.4 | 9.6 | 11.31 | 12.37 | 12.97 |  |
| ETV6 | 5.55 | 9.37 | 17.14 | 15.99 | 8.93 |  |
| SP1 | 20.54 | 26.11 | 17.59 | 17.12 | 11.78 |  |
| GATA2 | 0.52 | 12.82 | 15.96 | 17.59 | 9.82 |  |
| EGR1 | 4.69 | 4.08 | 11.73 | 17.7 | 0.06 |  |
| LYL1 | 0.2 | 9.79 | 29.69 | 27.73 | 36.86 |  |
| ERG | 0.04 | 3.49 | 36.11 | 39.93 | 61.28 |  |
| LMO2 | 0.69 | 23.7 | 75.24 | 71.06 | 10.78 |  |
| TGFB1 | 6.91 | 101.1 | 210.39 | 175.12 | 127.78 |  |
|  |  |  |  |  |  |  |
|  |  |  |  |  |  |  |
| **H small nuclear RNA** | | | | | |  |
| symbol | D0 | D1 | D2 | D3 | EPC |  |
| SNHG5 | 322.94 | 240.18 | 140.5 | 95.48 | 123.49 |  |
| SNRPB | 298.97 | 100.95 | 143.23 | 133 | 123.88 |  |
| SNRPN | 264.45 | 130.48 | 106.72 | 89.65 | 60.32 |  |
| SNORD86 | 233.9 | 64.85 | 0 | 0.31 | 27.31 |  |
| SNRPD2 | 233.7 | 98.81 | 124.65 | 91.76 | 122.51 |  |
| SNRPF | 177.86 | 100.62 | 58.58 | 46.26 | 39.69 |  |
| SNRPG | 153.67 | 108.36 | 73.7 | 60.6 | 62.4 |  |
| SNHG1 | 131.92 | 68.09 | 41.74 | 27.04 | 22.17 |  |
| SNRPD3 | 120.69 | 92.78 | 55.82 | 53.61 | 48.26 |  |
| SNRNP70 | 116.61 | 64.88 | 73.55 | 70.02 | 64.3 |  |
| SNRPC | 104.24 | 78.28 | 49.34 | 45.47 | 40.17 |  |
| SNHG6 | 98.39 | 77.04 | 44.63 | 33.84 | 43.96 |  |
| SNRNP200 | 90.87 | 49.83 | 80.97 | 62.42 | 44.28 |  |
| SNRPD1 | 85.08 | 50.24 | 37.92 | 34.66 | 25.6 |  |
| SNRPA1 | 83.83 | 54.6 | 28.73 | 30.68 | 29.95 |  |
| SNHG4 | 73.37 | 26.61 | 24.98 | 28.06 | 21.57 |  |
| SNRPA | 72.46 | 60.12 | 48.91 | 33.63 | 24.45 |  |
| SNHG16 | 71.55 | 33.24 | 34.26 | 30.08 | 54.27 |  |
| SNRNP40 | 64.65 | 41.71 | 27.13 | 23.7 | 25.76 |  |
| SNORD116-4 | 62.06 | 32.61 | 20.16 | 20.31 | 8.35 |  |
| SNRPE | 59.71 | 34.91 | 24.06 | 20.22 | 24.18 |  |
| SNORD29 | 55.7 | 6.23 | 0 | 0 | 17.86 |  |
| SNHG8 | 52.4 | 44.12 | 18.17 | 17.17 | 33.01 |  |
| SNRPB2 | 50.31 | 35.06 | 29.34 | 28.34 | 28.08 |  |
| SNHG7 | 48.42 | 21.78 | 23.55 | 16.71 | 27.1 |  |
| SNORD22 | 45.87 | 18.06 | 10.1 | 6 | 3.84 |  |
| SNORD36A | 44.87 | 30.1 | 5.05 | 0 | 23.98 |  |
| SNHG3 | 44.62 | 17.41 | 9.98 | 8.03 | 14.76 |  |
| SNORD80 | 41.03 | 5.16 | 20.79 | 15.42 | 9.87 |  |
| SNORD64 | 40.61 | 11.48 | 3.21 | 8.99 | 9.83 |  |
| SNORD116-2 | 35.02 | 8.32 | 3.37 | 0 | 0 |  |
| SNRNP25 | 31.46 | 25.56 | 18.24 | 18.38 | 15.05 |  |
| SNORD107 | 31.22 | 7.16 | 0 | 0 | 2.49 |  |
| SNORA40 | 29.59 | 13.89 | 7.99 | 23.72 | 13.28 |  |
| SNORD25 | 28.95 | 17.48 | 0 | 0 | 0 |  |
| SNN | 27.93 | 15.62 | 46.09 | 52.47 | 14.15 |  |
| SNORD30 | 26.4 | 10.62 | 5.35 | 0 | 0 |  |
| SNORA18 | 26.18 | 7.52 | 13.26 | 5.62 | 17.99 |  |
| SNHG15 | 25.77 | 7.21 | 6.13 | 6.51 | 5.17 |  |
| SNORD96A | 24.93 | 10.03 | 10.1 | 9.99 | 9.59 |  |
| SNORD12B | 24.11 | 5.39 | 0 | 0 | 5.15 |  |
| SNORD44 | 21.54 | 0 | 7.27 | 0 | 0 |  |
| SNORA62 | 21.3 | 1.53 | 3.08 | 4.57 | 0 |  |
| SNORD116-21 | 18.36 | 9.33 | 8.36 | 20.69 | 14.89 |  |
| SNORA4 | 17.77 | 3.58 | 7.2 | 14.25 | 1.71 |  |
| SNORD109A | 17.37 | 11.65 | 0 | 5.8 | 2.78 |  |
| SNORD109B | 17.37 | 11.65 | 0 | 5.8 | 2.78 |  |
| SNORA61 | 17.19 | 1.92 | 3.87 | 1.91 | 11.02 |  |
| SNORD12 | 16.62 | 0 | 0 | 3.33 | 0 |  |
| SNORA70 | 16.32 | 10.95 | 14.7 | 16.36 | 6.98 |  |
| SNORD74 | 14.96 | 0 | 0 | 5 | 0 |  |
| SNORD75 | 14.96 | 0 | 0 | 0 | 0 |  |
| SNORD23 | 14.55 | 4.88 | 0 | 0 | 0 |  |
| SNORD58B | 14.55 | 0 | 0 | 14.62 | 0 |  |
| SNORA8 | 13.94 | 7.01 | 7.06 | 6.99 | 6.71 |  |
| SNORD26 | 13.81 | 0 | 4.66 | 0 | 4.43 |  |
| SNORD116-6 | 13.59 | 0 | 0 | 0 | 0 |  |
| SNORA3 | 13.37 | 5.76 | 5.8 | 3.83 | 0 |  |
| SNORD47 | 13.13 | 8.81 | 4.44 | 8.78 | 0 |  |
| SNORA10 | 12.95 | 1.86 | 7.5 | 0 | 0 |  |
| SNORA76 | 12.95 | 0 | 3.75 | 0 | 3.56 |  |
| SNORA6 | 12.71 | 7.99 | 6.44 | 1.59 | 1.53 |  |
| SNORA63 | 12.69 | 0 | 7.35 | 12.72 | 6.98 |  |
| SNORD19B | 12.6 | 6.34 | 0 | 0 | 0 |  |
| SNORD83B | 12.6 | 3.17 | 6.38 | 22.09 | 6.06 |  |
| SNORA67 | 12.44 | 8.94 | 12.6 | 16.03 | 17.1 |  |
| SNORD116-20 | 12.43 | 6.21 | 3.12 | 8.83 | 0 |  |
| SNORD88B | 11.77 | 0 | 5.96 | 2.95 | 0 |  |
| SNORD116-14 | 11.73 | 12.49 | 0 | 0 | 0 |  |
| SNRNP27 | 11.61 | 6.65 | 11.62 | 11.9 | 13.24 |  |
| SNORA51 | 11.22 | 0 | 0 | 0 | 0 |  |
| SNORD16 | 11.22 | 2.82 | 0 | 2.81 | 8.09 |  |
| SNORD31 | 11.22 | 16.93 | 5.68 | 5.62 | 0 |  |
| SNORA33 | 11.1 | 3.72 | 0 | 1.85 | 1.78 |  |
| SNORA73A | 11.07 | 8.06 | 4.86 | 4.21 | 5.89 |  |
| SNORD18B | 10.56 | 0 | 0 | 0 | 5.08 |  |
| SNHG12 | 10.19 | 4 | 6.3 | 6.48 | 5.98 |  |
| SNORA81 | 10.11 | 0 | 2.56 | 2.53 | 2.43 |  |
| SNORD27 | 9.97 | 0 | 10.1 | 0 | 4.8 |  |
| SNORD57 | 9.97 | 0 | 0 | 0 | 0 |  |
| SNORD17 | 9.82 | 11.68 | 1.81 | 1.79 | 5.15 |  |
| SNORD14B | 9.79 | 3.28 | 3.31 | 0 | 3.14 |  |
| SNORD116-22 | 9.74 | 6.21 | 4.19 | 0 | 0 |  |
| SNORD116-17 | 9.46 | 0 | 0 | 0.74 | 0 |  |
| SNORD116-19 | 9.46 | 0 | 0 | 0.74 | 0 |  |
| SNORD116-24 | 9.29 | 15.59 | 12.55 | 9.31 | 5.95 |  |
| SNORA25 | 9.16 | 1.84 | 5.57 | 5.51 | 1.76 |  |
| SNORD83A | 9.13 | 6.12 | 3.08 | 3.05 | 0 |  |
| SNORD116-1 | 8.83 | 5.91 | 0 | 14.75 | 8.49 |  |
| SNORD14E | 8.16 | 0 | 0 | 0 | 0 |  |
| SNORD76 | 7.98 | 12.04 | 8.08 | 0 | 3.84 |  |
| SNORD60 | 7.64 | 0 | 0 | 0 | 0 |  |
| SNORA72 | 7.48 | 1.88 | 0 | 9.37 | 0 |  |
| SNORA21 | 7.4 | 1.86 | 1.87 | 0 | 0 |  |
| SNORA64 | 7.33 | 1.84 | 0 | 5.51 | 5.29 |  |
| SNRNP35 | 7.13 | 4.72 | 5.95 | 8.7 | 11.87 |  |
| SNIP1 | 7.07 | 3.33 | 6.39 | 5.64 | 6.33 |  |
| SNORD14D | 7.04 | 0 | 10.7 | 0 | 3.39 |  |
| SNHG9 | 6.97 | 1.17 | 3.53 | 5.24 | 6.71 |  |
| SNORD123 | 6.9 | 0 | 0 | 0 | 0 |  |
| SNORD14C | 6.9 | 0 | 3.5 | 0 | 0 |  |
| SNORD89 | 6.9 | 0 | 4.66 | 4.61 | 6.64 |  |
| SNORD54 | 6.65 | 0 | 0 | 0 | 0 |  |
| SNORD95 | 6.65 | 0 | 6.74 | 0 | 0 |  |
| SNORD10 | 6.41 | 1.61 | 8.12 | 3.21 | 1.54 |  |
| SNORD52 | 6.41 | 6.45 | 0 | 6.43 | 6.17 |  |
| SNORD116-28 | 6.3 | 3.17 | 0 | 0 | 0 |  |
| SNORD116-11 | 6.19 | 0 | 0 | 0 | 0 |  |
| SNORD116-13 | 6.19 | 3.11 | 6.27 | 0 | 0 |  |
| SNORD58A | 6.19 | 0 | 0 | 0 | 0 |  |
| SNORD3A | 5.95 | 1.66 | 0 | 3.98 | 0.48 |  |
| SNORD88A | 5.88 | 2.96 | 0 | 0 | 0 |  |
| SNORD88C | 5.88 | 0 | 0 | 0 | 0 |  |
| SNORD116-26 | 5.81 | 0 | 0 | 0 | 0 |  |
| SNORA1 | 5.73 | 1.92 | 0 | 3.83 | 1.84 |  |
| SNORA44 | 5.61 | 1.88 | 7.58 | 1.87 | 1.8 |  |
| SNORA66 | 5.55 | 3.72 | 0 | 0 | 0 |  |
| SNORA48 | 5.44 | 1.82 | 3.67 | 0 | 1.74 |  |
| SNORD38B | 5.44 | 0 | 0 | 0 | 0 |  |
| SNORA65 | 5.38 | 1.81 | 0 | 1.8 | 1.73 |  |
| SNRNP48 | 5.38 | 6.9 | 5.1 | 4.82 | 3.25 |  |
| SNORA71A | 5.28 | 0 | 0 | 0 | 0 |  |
| SNORD103A | 5.18 | 5.21 | 3.5 | 6.92 | 0 |  |
| SNORD103B | 5.18 | 5.21 | 3.5 | 6.92 | 0 |  |
| SNORD45B | 5.13 | 5.16 | 0 | 0 | 0 |  |
| SNORD49A | 5.13 | 0 | 5.2 | 10.28 | 4.93 |  |
| SNORD56 | 5.13 | 0 | 0 | 0 | 0 |  |
| SNORD102 | 4.99 | 5.02 | 0 | 0 | 0 |  |
| SNORD4A | 4.99 | 0 | 0 | 5 | 0 |  |
| SNORD65 | 4.85 | 19.52 | 0 | 9.72 | 4.67 |  |
| SNORD15A | 4.81 | 0 | 0 | 3.21 | 0 |  |
| SNORD1A | 4.72 | 0 | 0 | 0 | 0 |  |
| SNORD4B | 4.72 | 0 | 0 | 0 | 0 |  |
| SNORD55 | 4.72 | 0 | 4.79 | 0 | 0 |  |
| SNHG10 | 4.68 | 1.84 | 2.88 | 1.1 | 2.83 |  |
| SNORD110 | 4.6 | 4.63 | 4.66 | 0 | 0 |  |
| SNORD24 | 4.6 | 0 | 4.66 | 0 | 0 |  |
| SNORD100 | 4.49 | 0 | 0 | 0 | 0 |  |
| SNORD108 | 4.46 | 0 | 0 | 0 | 0 |  |
| SNORD45C | 4.27 | 4.3 | 0 | 0 | 0 |  |
| SNORA26 | 4.17 | 0 | 6.34 | 0 | 0 |  |
| SNORD12C | 4.17 | 0 | 0 | 0 | 0 |  |
| SNORD104 | 4.08 | 0 | 0 | 0 | 3.92 |  |
| SNORD79 | 3.99 | 0 | 0 | 4 | 0 |  |
| SNORD32A | 3.9 | 0 | 0 | 7.82 | 0 |  |
| SNORA56 | 3.86 | 0 | 0 | 0 | 0 |  |
| SNORD33 | 3.82 | 0 | 0 | 0 | 3.67 |  |
| SNORA45 | 3.78 | 1.9 | 5.74 | 5.68 | 0 |  |
| SNORA41 | 3.74 | 1.88 | 1.89 | 1.87 | 0 |  |
| SNORA5B | 3.74 | 0 | 1.89 | 0 | 0 |  |
| SNHG11 | 3.71 | 3.56 | 2.9 | 1.69 | 3.57 |  |
| SNORA13 | 3.7 | 0 | 1.87 | 1.85 | 0 |  |
| SNORD91B | 3.59 | 0 | 0 | 0 | 0 |  |
| SNORA2B | 3.55 | 0 | 0 | 0 | 0 |  |
| SNORD94 | 3.55 | 0 | 0 | 0 | 0 |  |
| SNORD35B | 3.45 | 17.36 | 0 | 0 | 3.32 |  |
| SNORD97 | 3.39 | 0 | 3.43 | 0 | 1.63 |  |
| SNORD58C | 3.32 | 0 | 0 | 0 | 9.59 |  |
| SNORA73B | 3.26 | 4.67 | 3.69 | 1.06 | 1.19 |  |
| SNORD116-25 | 3.08 | 0 | 3.14 | 0 | 0 |  |
| SNORD21 | 3.04 | 3.06 | 3.08 | 0 | 0 |  |
| SNORD125 | 2.99 | 3.01 | 6.06 | 11.99 | 0 |  |
| SNORD46 | 2.9 | 0 | 0 | 0 | 0 |  |
| SNORD116-8 | 2.86 | 0 | 0 | 0 | 0 |  |
| SNORD124 | 2.64 | 0 | 2.67 | 0 | 0 |  |
| SNORA17 | 2.24 | 0 | 0 | 5.09 | 5.3 |  |
| SNORA32 | 2.11 | 2.12 | 0 | 0 | 4.06 |  |
| SNORA77 | 2.02 | 0 | 2.04 | 4.04 | 0 |  |
| SNORA27 | 1.99 | 6.02 | 0 | 0 | 0 |  |
| SNORA28 | 1.99 | 0 | 0 | 0 | 0 |  |
| SNORA30 | 1.93 | 0 | 0 | 0 | 0 |  |
| SNORA31 | 1.91 | 1.92 | 1.93 | 11.48 | 0 |  |
| SNORA11 | 1.89 | 5.7 | 5.74 | 3.79 | 3.64 |  |
| SNORA24 | 1.89 | 1.9 | 3.83 | 0 | 0 |  |
| SNORA20 | 1.87 | 0 | 7.58 | 0 | 0 |  |
| SNORA68 | 1.85 | 0 | 0 | 7.42 | 0 |  |
| SNORA14A | 1.83 | 0 | 0 | 0 | 0 |  |
| SNORA22 | 1.83 | 1.84 | 0 | 3.67 | 0 |  |
| SNORA42 | 1.83 | 0 | 0 | 0 | 0 |  |
| SNORA52 | 1.83 | 1.84 | 1.86 | 0 | 1.76 |  |
| SNORA14B | 1.81 | 0 | 0 | 1.82 | 0 |  |
| SNORA2A | 1.81 | 1.82 | 0 | 0 | 0 |  |
| SNORA50 | 1.79 | 0 | 0 | 0 | 0 |  |
| SNORA75 | 1.78 | 0 | 0 | 0 | 0 |  |
| SNORA71D | 1.76 | 0 | 0 | 0 | 0 |  |
| SNORA23 | 1.17 | 0 | 0 | 1.18 | 0 |  |
| SNORA74A | 1.09 | 1.1 | 0 | 0 | 1.05 |  |
| SNORA58 | 0.9 | 0 | 0.91 | 2.7 | 0 |  |
| SNORA53 | 0.84 | 2.53 | 0.85 | 0.84 | 1.61 |  |
| SNORD73A | 0.15 | 0 | 1.26 | 0 | 0 |  |
| SNORA11D | 0 | 0 | 1 | 0 | 0 |  |
| SNORA11E | 0 | 0 | 1 | 0 | 0 |  |
| SNORA16A | 0 | 1.84 | 1.86 | 1.84 | 0 |  |
| SNORA16B | 0 | 0 | 0 | 1.85 | 1.78 |  |
| SNORA34 | 0 | 1.79 | 0 | 1.78 | 0 |  |
| SNORA36B | 0 | 1.9 | 0 | 0 | 0 |  |
| SNORA46 | 0 | 0 | 1.84 | 0 | 0 |  |
| SNORA55 | 0 | 0 | 1.8 | 0 | 1.71 |  |
| SNORA57 | 0 | 0 | 1.61 | 0 | 4.58 |  |
| SNORA59A | 0 | 0 | 0.78 | 1.55 | 0.74 |  |
| SNORA59B | 0 | 0 | 0.78 | 1.55 | 0.74 |  |
| SNORA5A | 0 | 0 | 1.86 | 0 | 0 |  |
| SNORA5C | 0 | 1.79 | 1.8 | 3.56 | 1.71 |  |
| SNORA60 | 0 | 0 | 1.82 | 0 | 0 |  |
| SNORA71B | 0 | 1.81 | 0 | 3.6 | 0 |  |
| SNORA71C | 0 | 0 | 0 | 1.76 | 0 |  |
| SNORA7A | 0 | 3.51 | 0 | 0 | 0.53 |  |
| SNORA7B | 0 | 0 | 0 | 0 | 0.53 |  |
| SNORD113-2 | 0 | 0 | 0 | 0 | 9.87 |  |
| SNORD116-12 | 0 | 0 | 3.14 | 0 | 0 |  |
| SNORD116-23 | 0 | 3.09 | 0 | 0 | 0 |  |
| SNORD116-29 | 0 | 0 | 3.71 | 0 | 0 |  |
| SNORD116-3 | 0 | 5.18 | 1.49 | 2.95 | 0 |  |
| SNORD116-5 | 0 | 5.18 | 1.49 | 0 | 0 |  |
| SNORD116-7 | 0 | 5.18 | 1.49 | 0 | 0 |  |
| SNORD116-9 | 0 | 5.18 | 1.49 | 2.95 | 0 |  |
| SNORD118 | 0 | 1.03 | 0 | 0 | 0 |  |
| SNORD121B | 0 | 4.2 | 4.23 | 8.37 | 0 |  |
| SNORD15B | 0 | 0 | 0 | 0 | 1.57 |  |
| SNORD2 | 0 | 0 | 0 | 0 | 5.08 |  |
| SNORD20 | 0 | 4.1 | 0 | 0 | 0 |  |
| SNORD28 | 0 | 0 | 4.66 | 0 | 0 |  |
| SNORD35A | 0 | 0 | 0 | 3.6 | 0 |  |
| SNORD36B | 0 | 0 | 0 | 0 | 29.6 |  |
| SNORD36C | 0 | 5.64 | 0 | 0 | 0 |  |
| SNORD37 | 0 | 6.02 | 0 | 0 | 5.76 |  |
| SNORD38A | 0 | 0 | 0 | 0 | 4.93 |  |
| SNORD3B-1 | 0 | 1.66 | 0 | 0 | 0.48 |  |
| SNORD3B-2 | 0 | 1.66 | 0 | 0 | 0.48 |  |
| SNORD3C | 0 | 0 | 0 | 0 | 0.48 |  |
| SNORD41 | 0 | 0 | 0 | 5.29 | 0 |  |
| SNORD42B | 0 | 0 | 0 | 0 | 5.57 |  |
| SNORD45A | 0 | 0 | 0 | 3.75 | 3.6 |  |
| SNORD50B | 0 | 0 | 0 | 5.14 | 0 |  |
| SNORD53 | 0 | 0 | 0 | 4.28 | 0 |  |
| SNORD59B | 0 | 4.63 | 0 | 0 | 0 |  |
| SNORD6 | 0 | 10.32 | 0 | 0 | 4.93 |  |
| SNORD62A | 0 | 1.81 | 1.82 | 7.2 | 3.45 |  |
| SNORD62B | 0 | 1.81 | 1.82 | 7.2 | 3.45 |  |
| SNORD63 | 0 | 5.64 | 0 | 0 | 0 |  |
| SNORD68 | 0 | 0 | 5.05 | 14.99 | 14.39 |  |
| SNORD69 | 0 | 4.4 | 0 | 0 | 4.21 |  |
| SNORD72 | 0 | 4.1 | 0 | 0 | 0 |  |
| SNORD82 | 0 | 0 | 0 | 0 | 4.43 |  |
| SNORD87 | 0 | 0 | 4.55 | 0 | 0 |  |
| SNORD99 | 0 | 0 | 0 | 4.09 | 0 |  |
