# Supplementary material for: FLI1 and PKC co-activation promote highly efficient differentiation of human embryonic stem cells into endothelial-like cells
Source: Cell Death Dis. 2018 Jan 26;9(2):131. doi: 10.1038/s41419-017-0162-9 (PMC5833666; doi:10.1038/s41419-017-0162-9)
Supplement: Supplementary file 4 — primers sequences [file 41419_2017_162_MOESM4_ESM.docx]

|  |  |  |  |  |
| --- | --- | --- | --- | --- |
|  | **Table 2 primers sequences** | | |  |
|  |  |  |  |  |
|  | **Name** | **Forward** | **Reverse** | **Length (BP)** |
|  | OCT4 | CTTGCTGCAGAAGTGGGTGGAGGAA | CTGCAGTGTGGGTTTCGGGCA | 169 |
|  | SOX2 | AGCTACAGCATGATGCAGGA | GGTCATGGAGTTGTACTGCA | 126 |
|  | NANOG | AAGGTCCCGGTCAAGAAACAG | CTTCTGCGTCACACCATTGC | 237 |
|  | KLF4 | TCTCAAGGCACACCTGCGAA | TAGTGCCTGGTCAGTTCATC | 105 |
|  | CMY-C | GTCAAGAGGCGAACACACAAC | TTGGACGGACAGGATGTATGC | 162 |
|  | PAX6 | AACGATAACATACCAAGCGTGT | GGTCTGCCCGTTCAACATC | 120 |
|  | NESTIN | AAGATGTCCCTCAGCCTGG | GAGGGAAGTCTTGGAGCCAC | 99 |
|  | KRT17 | GGAGATTGCCACCTACCG | TGCCATCCTGGACCTCTT | 119 |
|  | SOX1 | TCAAACGGCCCATGAACGC | CGGCCTCGGACATGACCTTC | 135 |
|  | BRACHYURY(T) | TGCTTCCCTGAGACCCAGTT | GATCACTTCTTTCCTTTGCATCAAG | 121 |
|  | MIXL1 | CCGAGTCCAGGATCCAGGTA | CTCTGACGCCGAGACTTGG | 58 |
|  | AFP | GGGAGCGGCTGACATTAT | TGTTTCATCCACCACCAA | 139 |
|  | SOX17 | CAGTGACGACCAGAGCCAGACC | CCACGACTTGCCCAGCATCTT | 292 |
|  | FOX2A | ATGCACTCGGCTTCCAGTAT | CATGTACGTGTTCATGCCGT | 150 |
|  | CXCR4 | CACCGCATCTGGAGAACCA | GCCCATTTCCTCGGTGTAGTT | 178 |
|  | CD144 | GTTCACGCATCGGTTGTTCAA | CGCTTCCACCACGATCTCATA | 238 |
|  | CD31 | AACAGTGTTGACATGAAGAGCC | TGTAAAACAGCACGTCATCCTT | 148 |
|  | CD34 | CTACAACACCTAGTACCCTTGGA | GGTGAACACTGTGCTGATTACA | 185 |
|  | FLK-1 | GGTATTGGCAGTTGGAGGAA | ACATTTGCCGCTTGGATAAC | 199 |
|  | vWF | AGCCTTGTGAAACTGAAGCAT | GCCCTGGTTGCCATTGTAATTC | 237 |
|  | CD133 | AGTCGGAAACTGGCAGATAGC | GGTAGTGTTGTACTGGGCCAAT | 99 |
|  | VEGFA | AGGGCAGAATCATCACGAAGT | AGGGTCTCGATTGGATGGCA | 75 |
|  | GATA2 | GCAACCCCTACTATGCCAACC | CAGTGGCGTCTTGGAGAAG | 212 |
|  | CD90 | ATCGCTCTCCTGCTAACAGTC | CTCGTACTGGATGGGTGAACT | 135 |
|  | Fli1 | CCAACGAGAGGAGAGTCATCG | TTCCGTGTTGTAGAGGGTGGT | 200 |
|  | β-Actin | CGCACCACTGGCATTGTCAT | TTCTCCTTGATGTCACGCAC | 206 |
