# Supplementary material for: FLI1 and PKC co-activation promote highly efficient differentiation of human embryonic stem cells into endothelial-like cells
Source: Cell Death Dis. 2018 Jan 26;9(2):131. doi: 10.1038/s41419-017-0162-9 (PMC5833666; doi:10.1038/s41419-017-0162-9)
Supplement: Supplementary file 5 — Supplementary Figure Legends [file 41419_2017_162_MOESM5_ESM.docx]

**Supplementary Figure Legends**

**Supplementary Figure 1.** hESC and hiPSC cell lines expressed pluripotent markers. hESC cell lines (hESC-137 and hESC-254) and hiPSC cell lines (SF-iPS and UC013) all expressed pluripotent markers SOX2, OCT4 and TRA-1-60. Scale bar, 50 μm.

**Supplementary Figure 2.** *FLI1* and PKC co-activation mediated hiPSCs differentiation into iECs. **(A)**
